# Computer-Aided Classification Framework of Parkinsonian Disorders Using ^11^C-CFT PET Imaging

**DOI:** 10.3389/fnagi.2021.792951

**Published:** 2022-02-01

**Authors:** Jiahang Xu, Qian Xu, Shihong Liu, Ling Li, Lei Li, Tzu-Chen Yen, Jianjun Wu, Jian Wang, Chuantao Zuo, Ping Wu, Xiahai Zhuang

**Affiliations:** ^1^PET Center, Huashan Hospital, Fudan University, Shanghai, China; ^2^School of Data Science, Fudan University, Shanghai, China; ^3^School of Computer Science, University of Nottingham, Nottingham, United Kingdom; ^4^School of Biomedical Engineering, Shanghai Jiao Tong University, Shanghai, China; ^5^Nuclear Medicine and Molecular Imaging Center, Linkou Chang Gung Memorial Hospital, Chang Gung University, Taoyuan, Taiwan; ^6^Department of Neurology, Huashan Hospital, Fudan University, Shanghai, China; ^7^National Center for Neurological Disorders & National Clinical Research Center for Aging and Medicine, Huashan Hospital, Fudan University, Shanghai, China; ^8^Human Phenome Institute, Fudan University, Shanghai, China; ^9^Institute of Functional and Molecular Medical Imaging, Fudan University, Shanghai, China

**Keywords:** ^11^C-CFT PET imaging, computer-aided diagnosis, Parkinson's disease, multiple system atrophy, progressive supranuclear palsy

## Abstract

**Purpose:**

To investigate the usefulness of a novel computer-aided classification framework for the differential diagnosis of parkinsonian disorders (PDs) based on ^11^C-methyl-N-2β-carbomethoxy-3β-(4-fluorophenyl)-tropanel (^11^C-CFT) positron emission tomography (PET) imaging.

**Methods:**

Patients with different forms of PDs—including Parkinson's disease (PD), multiple system atrophy (MSA) and progressive supranuclear palsy (PSP)—underwent dopamine transporter (DAT) imaging with ^11^C-CFT PET. A novel multistep computer-aided classification framework—consisting of magnetic resonance imaging (MRI)-assisted PET segmentation, feature extraction and prediction, and automatic subject classification—was developed. A random forest method was used to assess the diagnostic relevance of different regions to the classification process. Finally, the performance of the computer-aided classification system was tested using various training strategies involving patients with early and advanced disease stages.

**Results:**

Accuracy values for identifying PD, MSA, and PSP were 85.0, 82.2, and 89.7%, respectively—with an overall accuracy of 80.4%. The caudate and putamen provided the highest diagnostic relevance to the proposed classification framework, whereas the contribution of midbrain was negligible. With the exception of sensitivity for diagnosing PSP, the strategy comprising both early and advanced disease stages performed better in terms of sensitivity, specificity, positive predictive value, and negative predictive value within each PDs subtype.

**Conclusions:**

The proposed computer-aided classification framework based on ^11^C-CFT PET imaging holds promise for improving the differential diagnosis of PDs.

## Introduction

Parkinsonian disorders (PDs) are a heterogeneous group of neurological disorders characterized by tremor, bradykinesia, rigidity, and poor postural stability. Parkinson's disease (PD)—the most common PDs occurring in the elderly (Pringsheim et al., 2014)—should be differentiated by other atypical parkinsonian syndromes (APSs), including multiple system atrophy (MSA) and progressive supranuclear palsy (PSP) (Fahn et al., 2011). A correct differential diagnosis between PD, MSA, and PSP is paramount not only for proper treatment allocation and prognostication, but also for correct implementation of clinical trials focusing on disease-modifying drugs. Unfortunately, an accurate diagnosis remains challenging both because of a consistent overlap of signs and symptoms and the presence of atypical manifestations—especially in early disease stages (Steele et al., 1964). The key pathogenic mechanisms of PDs consist of a progressive neuronal loss in the substantia nigra of midbrain (Burns et al., 1983) and the occurrence of dopaminergic dysfunction in the striatum (Hantraye et al., 1992; Strafella et al., 2017). Although PDs were found to have different midbrain neuronal loss and striatal dopaminergic dysfunction patterns, their effect in PDs classification remains to be discussed (Martin-Bastida et al., 2017).

DAT PET imaging is clinically useful to differentiate PDs from conditions unrelated to dopaminergic dysfunction (e.g., essential tremor and drug-induced or psychogenic parkinsonism) (Benamer et al., 2000; Marshall et al., 2006; Thobois et al., 2019). However, its use in differentiation among PDs remains elusive. Knudsen et al. (2004) demonstrated that patients with PD have significantly higher striatal asymmetry than those with MSA, whereas Ilgin and coworkers (Ilgin et al., 1999) reported that PD is characterized by a more pronounced loss of DAT in the posterior putamen compared with PSP. However, studies have shown that regional analysis of DAT PET images does not allow differentiating between PD and MSA (Pirker et al., 2000; Varrone et al., 2001; Perju-Dumbrava et al., 2012). Therefore, in current practice, patients considered with PDs frequently undergo two different PET protocols, i.e., DAT PET for excluding disorders unrelated to dopaminergic dysfunction and ^18^F-FDG PET for PDs differentiation (Zhao et al., 2012), which is costly and time-consuming.

Recently, computer-aided medical diagnosis has emerged as a valuable tool to extract information from medical images in the context of early-stage PDs (Teune et al., 2014; Tripathi et al., 2016; Matthews et al., 2018; Xu et al., 2019). In this scenario, this study was undertaken to investigate the clinical utility of a novel computer-aided classification framework for PDs using ^11^C-methyl-N-2β-carbomethoxy-3β-(4-fluorophenyl)-tropanel (^11^C-CFT) DAT PET imaging.

## Materials and Methods

### Subjects

A total of 107 patients with PDs were recruited from the Department of Neurology, Huashan Hospital, Fudan University (Shanghai, China). Another group of 22 normal controls (NC) was recruited as reference for PDs patients in the aspect of DAT binding level. All participants were screened by two experts in the field of movement disorders before PET imaging. Follow-up was continued for at least one year. The clinical diagnosis of “definite” PD was made according to the UK Brain Bank criteria (Hughes et al., 1992) and confirmed using the Movement Disorder Society clinical diagnostic criteria (Postuma et al., 2015). All of the patients with MSA and PSP conformed to the second consensus statement on the diagnosis of “probable” MSA (Gilman et al., 2008) and the consensus diagnostic criteria for “probable” PSP (NINDS-SPSP) (Litvan et al., 1996), respectively. Exclusion criteria were as follows: history of other neurological or psychiatric disorders; use of neuroleptics; and presence of structural brain lesions on MRI (i.e., masses, white matter changes, ischemia, or hemorrhage). The UPDRS motor examination (items 18–31) and the Hoehn and Yahr (H&Y) stage were determined within 2 h of PET imaging and at least 12 h after discontinuation of antiparkinsonian medications (Wu et al., 2013). The study patients were divided into patients with early-stage (ES) and advanced-stage (AS) disease (duration threshold: 24 months). Ethical approval was granted by the Institutional Review Board of the Huashan Hospital and written consent was obtained from all participants.

### Data Acquisition

PET images were acquired using a Siemens Biograph 64 PET/CT scanner (Siemens, Munich, Germany) in the three-dimensional (3D) mode. A computed tomography (CT) transmission scan was initially performed for attenuation correction. A PET scan of 15 min was started 60 min after the intravenous injection of 370 MBq of ^11^C-CFT. During scanning, patients laid comfortably in supine position in a room with dimmed lighting and low background noise (Huang et al., 2020). MRI data were acquired using the T1-weighted 3D inversion recovery spoiled gradient recalled acquisition (IR-SPGR) technique, as previously described (Bu et al., 2018). PET and MRI scans were performed within one week of each other.

### Diagnostic Methods

**Figure 1:**
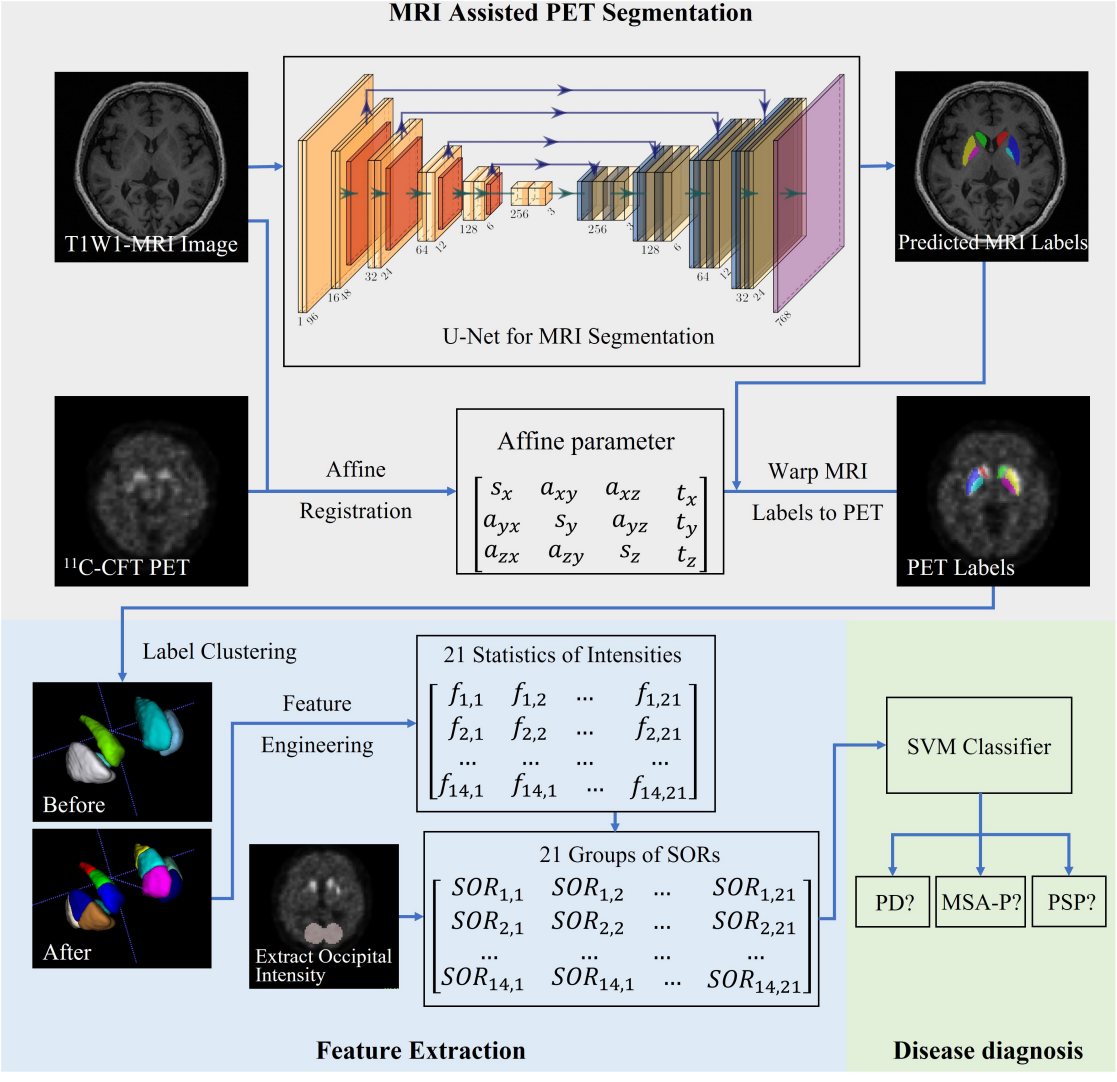
Visual representation of the proposed computer-aided classification framework. Magnetic resonance imaging (MRI)-assisted ^11^C-CFT PET segmentation was followed by feature extraction and prediction. The final step consisted of automatic subject classification in each of the three diagnostic groups (PD, MSA, and PSP).

Figure 1 provides a workflow of the proposed multistep computer-aided classification framework—which consisted of MRI-assisted PET segmentation, feature extraction and prediction, and automatic subject classification. The detailed procedures have been previously described (Xu et al., 2019).

#### MRI-Assisted PET Segmentation and Feature Extraction

A U-net was implemented to carry out segmentation analysis of the striatum—including the bilateral caudate, putamen, pallidus and midbrain—on MRI images. Bilateral occipital areas were segmented using the ITK-SNAP software package. The U-net—which consists of an encoder-decoder network with skip connections—is based on an optimized convolutional neural network (CNN) (Ronneberger et al., 2015; Wong et al., 2018). Segmented MRI images were thoroughly aligned to the corresponding PET images by traditional registration methods using the C++ programming language, and the resulting transformation matrix was subsequently used to transform the label information of the MRI image onto the PET image (Xu et al., 2019). Based on the segmentation results, the bilateral caudate and putamen were further divided into three subregions with an equal volume (anterior, middle, and posterior) achieved by a k-means clustering algorithm in Python (Ng et al., 2006). Features were extracted by determining the volumes of tracer uptake from a total of 16 subregions. The striatal-to-occipital ratio (SOR) of the extracted features was calculated for normalization purposes.

#### SVM Classification Analysis

Machine learning of the extracted features and diagnostic classifications were implemented using the software machine learning library Scikit-learn 0.22.0 (https://pypi.org/project/scikit-learn). The selection of the most suitable classifier was performed by comparing the diagnostic performances with various algorithms on the specified dataset—including SVM (Haller et al., 2012; Long et al., 2012), logistic regression (Tripathi et al., 2016) and random forest algorithm (Gray et al., 2013). Owing to its capacity to handle high-dimensional feature spaces with high efficiency, a linear SVM classifier with squared hinge loss was selected for performing the classification task. All of the parameters of the SVM solver were optimized for subsequent experiments.

#### Training Strategies for Early-Stage Patients

The ES and AS groups consisted of 36 and 71 patients, respectively. The classification performances in different subsetting of ES and AS patients was tested using four strategies, as follows: (1) 71 AS + 35 ES/1 ES (training on both the ES and AS groups, and test on the ES group with a leave-one-out cross-validation strategy); (2) 35 ES/1 ES (training and testing on the ES group with a leave-one-out cross-validation strategy); (3) 71 AS/36 ES (training on the AS group and testing on the ES group); and (4) 36 AS/36 ES (training and testing on the AS group, with the size of the training set made equal to the ES group).

#### Statistical Analysis

We assessed demographic and clinical data with ANOVA, Kruskal-Wallis, and χ^2^ tests, as appropriate. The accuracy, sensitivity, specificity, positive predictive value (PPV), and negative predictive value (NPV) for the binary and multiclass classification of each PDs was determined. Areas under the receiver operating characteristic (ROC) curve (AUCs) were calculated to assess the diagnostic accuracy. The potential added value of the inclusion of midbrain for the purpose of multiclass classification was assessed using the McNemar's test. All above analyses were carried out in GraphPad Prism, version 8.0.1 (Graph Pad Inc., San Diego, CA, USA). Statistical significance was determined by a two-tailed *p* value < 0.05. The relative contribution of each striatal subregion to the classification process was determined using the random forest method based on Python. and visualized with the BrainNet Viewer (Xia et al., 2013).

## Results

### Subjects

Of the 107 participants, 50, 37, and 20 were clinically diagnosed with PD, MSA, and PSP, respectively. Demographic and clinical characteristics of subjects and normal controls are shown in Table 1.

**Table 1:** General characteristics of the study patients (*n* = 107) and normal controls (NC) (*n* = 22).

	**PD**	**MSA**	**PSP**	**NC**
Disease stage, ES%	22.0%	45.9%	40.0%	–
Sex, male%	64.0%	67.6%	80.0%	77.7%
Age, years	61.54 ± 7.41	59.24 ± 7.58	65.00 ± 8.32	64.91 ± 6.91
Disease duration, years	3.53 ± 1.99	2.32 ± 1.37	2.82 ± 1.67	–
UPDRS-III	21.94 ± 9.69	30.13 ± 14.42	23.90 ± 12.87	–
Dose equivalent, mg	275.8 ± 301.0	267.5 ± 329.9	161.3 ± 196.9	–

*Data are given as counts or means ± standard deviations. PD, Parkinson's disease; MSA, multiple system atrophy; PSP, progressive supranuclear palsy; ES, early-stage; UPDRS-III, Unified Parkinson's Disease Rating Scale – part III*.

**Figure 2:**
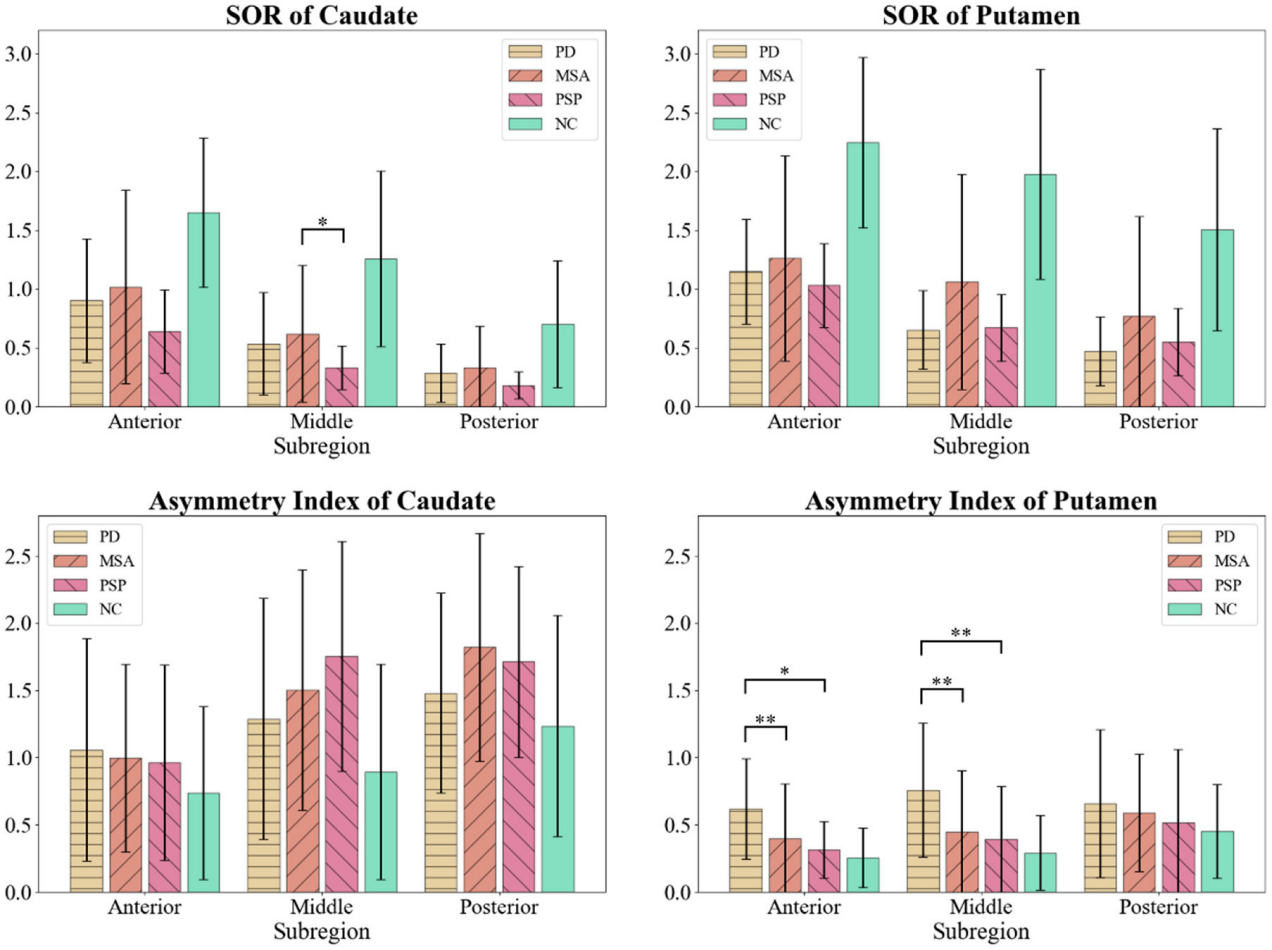
Comparison of SOR values and bilateral asymmetric indexes among parkinsonism groups and normal controls (NC). *p < 0.05; **p < 0.01. SOR value of middle caudate was lower in PSP group than MSA group. Asymmetric indexes of anterior and middle putamen were elevated in PD group compared with all other groups.

Detailed DAT distribution of patient groups are shown in Table 2; Figure 2. Average SOR values of both sides of subregions, and asymmetric indexes [(SOR of the higher side) - (SOR of the lower side)] / (average SOR of both sides) were calculated out and compared. Compared with NC group, patient groups revealed a significant decline of SOR values. However, almost no significant difference was found among parkinsonism groups. Of note, asymmetric indexes of anterior and middle putamen were significantly elevated in PD group compared with all the other groups. At the same time, MSA and PSP group performed higher asymmetric indexes in middle caudate than NC subjects.

**Table 2:** Average SOR values and bilateral asymmetric indexes for parkinsonism groups and normal controls (NC).

					***p*** **value**					
	**PD**	**MSA**	**PSP**	**NC**	**PD vs. MSA**	**PD vs. PSP**	**MSA vs. PSP**	**PD vs. NC**	**MSA vs. NC**	**PSP vs. NC**
**SOR values**										
Anterior caudate	0.90 ± 0.53	1.02 ± 0.83	0.58 ± 0.40	1.65 ± 0.65	ns	ns	ns	***	**	***
Middle caudate	0.52 ± 0.44	0.62 ± 0.59	0.25 ± 0.22	1.26 ± 0.77	ns	ns	*	***	**	***
Posterior caudate	0.25 ± 0.25	0.32 ± 0.36	0.14 ± 0.13	0.70 ± 0.55	ns	ns	ns	***	*	***
Anterior putamen	1.13 ± 0.48	1.26 ± 0.88	1.03 ± 0.37	2.25 ± 0.74	ns	ns	ns	***	***	***
Middle putamen	0.65 ± 0.34	1.06 ± 0.93	0.67 ± 0.29	1.98 ± 0.91	ns	ns	ns	***	**	***
Posterior putamen	0.46 ± 0.30	0.77 ± 0.86	0.55 ± 0.29	1.50 ± 0.88	ns	ns	ns	***	*	**
**Asymmetric index**										
Anterior caudate	1.06 ± 0.84	1.00 ± 0.71	0.97 ± 0.71	0.74 ± 0.66	ns	ns	ns	ns	ns	ns
Middle caudate	1.28 ± 0.90	1.50 ± 0.91	1.57 ± 0.83	0.89 ± 0.82	ns	ns	ns	ns	*	*
Posterior caudate	1.41 ± 0.72	1.78 ± 0.86	1.54 ± 0.71	1.24 ± 0.84	ns	ns	ns	ns	ns	ns
Anterior putamen	0.63 ± 0.38	0.40 ± 0.41	0.31 ± 0.22	0.25 ± 0.23	**	*	ns	***	ns	ns
Middle putamen	0.76 ± 0.50	0.45 ± 0.47	0.39 ± 0.40	0.29 ± 0.28	**	**	ns	***	ns	ns
Posterior putamen	0.67 ± 0.55	0.59 ± 0.44	0.52 ± 0.56	0.45 ± 0.36	ns	ns	ns	ns	ns	ns

*Data are given as means ± standard deviations. PD, Parkinson's disease; MSA, multiple system atrophy; PSP, progressive supranuclear palsy; ns, no significant difference*;

* *p < 0.05*,

** *p < 0.01*,

*** *p < 0.001*.

### Binary and Multiclass Classification of Parkinsonian Disorders

The goal of binary classification was to discriminate between one subtype of PDs and the remaining two. The accuracy values of this classification for distinguishing PD, MSA, and PSP from the other two diagnostic groups were 85.0, 82.2, and 89.7% respectively. The AUCs from ROC curve analysis were 85.0, 80.1, and 82.1% respectively. The sensitivity, specificity, PPV, and NPV for the diagnosis are shown in Table 3.

**Table 3:** Diagnostic performances of the proposed computer-aided classification framework for the binary classification of different parkinsonian disorders.

**Classification group**	**Accuracy**	**AUC**	**Sensitivity**	**Specificity**	**PPV**	**NPV**
PD vs. others	85.0%	85.0%	84.0%	86.0%	84.0%	86.0%
MSA vs. others	82.2%	80.1%	73.0%	87.1%	75.0%	85.9%
PSP vs. others	89.7%	82.1%	70.0%	94.3%	73.7%	93.2%

*PD, Parkinson's disease; MSA, multiple system atrophy; PSP, progressive supranuclear palsy; AUC, area under curve; PPV, positive predictive value; NPV, negative predictive value*.

The overall accuracy of multiclass classification—defined as the proportion of instances being correctly predicted across all study participants—was 80.4%. The sensitivity and specificity of multiclass classification are shown in Table 4. The NPV was similar for the three diagnostic categories (~90%), whereas the PPV was higher for PD (Table 3).

**Table 4:** Confusion matrix and diagnostic performances of the multi-class classification system of different parkinsonian disorders.

**SVM classification**	**Diagnostic categories (SOT)**			**PPV/NPV**
	**PD**	**MSA**	**PSP**	
PD	**43**	5	4	82.7%/87.3%
MSA	6	**29**	2	78.4%/88.6%
PSP	1	3	**14**	77.7%/93.3%
Sensitivity/specificity	86.0%/84.2%	78.4%/88.6%	70.0%/95.4%	

*SVM, support vector machine; SOT, standard of truth; PD, Parkinson's disease; MSA, multiple system atrophy; PSP, progressive supranuclear palsy; PPV, positive predictive value; NPV, negative predictive value. The number of true positive subjects are marked in bold*.

### Diagnostic Results According to Different Training Strategies

We next investigated the classification accuracies for ES patients using the above-mentioned four different training strategies. Strategy 1 resulted in the highest accuracy (77.8%), which was found to decrease in a stepwise fashion from strategy 1 to strategy 4 (Table 5). The sensitivity, specificity, PPV and NPV obtained with the application of strategy 1 were 90.9, 80.0, 66.7, and 95.2%, respectively, for the diagnosis of PD; 88.2, 84.2, 83.3, and 88.9%, respectively, for the diagnosis of MSA; and 37.5, 100.0, 100.0, and 84.9%, respectively, for the diagnosis of PSP.

**Table 5:** Diagnostic performances of the four different training strategies.

**Strategy (training/testing)**	**Accuracy**	**Error count (errors/total)**	**Diagnostic group**	**Sensitivity**	**Specificity**	**PPV**	**NPV**
1 71 AS+ 35 ES/1 ES	**77.8%**	8/36	PD	**90.9%**	80.0%	66.7%	**95.2%**
			MSA	**88.2%**	**84.2%**	**83.3%**	**88.9%**
			PSP	37.5%	**100.0%**	**100.0%**	**84.9%**
2 35 ES/1 ES	69.4%	11/36	PD	63.6%	**88.0%**	**70.0%**	84.6%
			MSA	76.5%	79.0%	76.5%	79.0%
			PSP	**62.5%**	85.7%	55.6%	88.9%
371 AS/36 ES	58.3%	15/36	PD	54.5%	76.0%	50.0%	79.2%
			MSA	82.4%	57.9%	63.6%	78.6%
			PSP	37.5%	96.4%	50.0%	79.4%
435 AS/36 ES	55.6%	16/36	PD	54.6%	76.0%	50.0%	79.2%
			MSA	70.6%	57.9%	60.0%	68.7%
			PSP	25.0%	92.9%	50.0%	81.3%

*PPV, positive predictive value; NPV, negative predictive value; AS, advanced stage; ES, early stage; PD, Parkinson's disease; MSA, multiple system atrophy; PSP, progressive supranuclear palsy. The strategy column reports different combinations of training subjects while maintaining test subjects constant. Best performing values are marked in bold*.

### Relative Contribution of Subregional Features to the Diagnostic Classification

**Figure 3:**
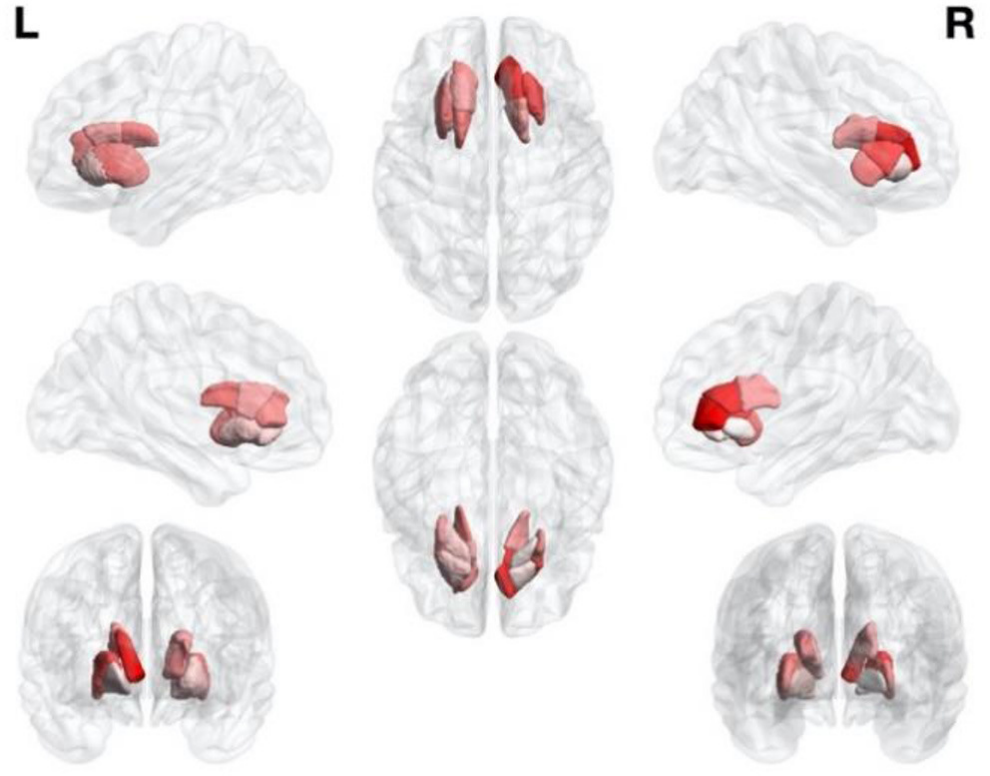
Relative contribution of different components of the striatum to the proposed computer-aided classification framework. Intensity of colors reflects the magnitude of contribution.

The caudate contributed more prominently (49.2%) than the putamen (41.6%) and pallidum (9.2%) to the classification process. Specifically, anterior caudate (18.5%), middle putamen (16.8%), and middle caudate (16.3%) contributed more than posterior putamen (14.7%), posterior caudate (14.4%), anterior putamen (10.1%), and pallidum (9.2%). The relative contributions of different striatal subregions to the diagnostic classification are summarized in Table 6 and visualized with the BrainNet Viewer (Figure 3) and. The addition of the midbrain resulted in a slight improvement in terms of accuracy (1.8%), without a significant effect (*p* = 0.683) on multiclass classification. Training based on midbrain alone resulted in a poor diagnostic accuracy (48.6%) (Table 7).

**Table 6:** Relative contribution of different regions to the computer-aided classification framework.

**Region**	**Contribution**	**Region**	**Contribution**
Anterior caudate (R)	0.113	Anterior caudate (L)	0.072
Middle caudate (R)	0.099	Posterior caudate (R)	0.067
Middle putamen (R)	0.092	Middle caudate (L)	0.064
Posterior caudate (L)	0.077	Pallidum (L)	0.054
Middle putamen (L)	0.076	Anterior putamen (L)	0.053
Posterior putamen (R)	0.075	Anterior putamen (R)	0.048
Posterior putamen (L)	0.072	Pallidum (R)	0.038

*R, right; L, left. Diagnostic characteristics were not adjusted. The sum of all relative contribution values is 1*.

**Table 7:** Accuracy of different combinations of regions of interest.

**Strategy**	**Midbrain + striatum**	**Midbrain**	**Striatum**	***p*** **value**
Accuracy	82.2%	48.6%	80.4%	0.683

*(1) Midbrain + striatum: the classifier was trained using features extracted from both midbrain and striatum; (2) Midbrain: the classifier was trained using features extracted only from midbrain; (3) Striatum only: the classifier was trained using features extracted only from striatum. p value refers to the difference between the analysis with the extracted features from midbrain + striatum and striatum*.

## Discussion

In the current study, we demonstrate the feasibility of a novel computer-aided classification framework for PDs based on ^11^C-CFT PET imaging. Specifically, the accuracy values of the proposed strategy for identifying PD, MSA, and PSP were 85.0, 82.2, and 89.7%, respectively—with an overall accuracy in the context of PDs of 80.4%. The caudate and putamen had the highest diagnostic relevance within the proposed classification strategy, whereas the contribution of the midbrain was negligible. If independently validated, our results may set the stage to the application of the proposed computer-aided classification approach for distinguishing among PD, MSA, and PSP.

While PD is generally characterized by a relatively preserved DAT binding in the caudate during early disease stages followed by a rostrocaudal progression in dopaminergic denervation, diffuse striatal impairment is typical of both MSA and PSP (Georgiopoulos et al., 2015). A recent meta-analysis provided a comprehensive comparison of striatal presynaptic dopaminergic function across the spectrum of PDs (Kaasinen et al., 2019). Compared with PD and MSA, PSP is characterized by the most pronounced loss of DAT function at both caudate and putamen; in addition, caudate DAT binding is lower in patients with MSA than in those with PD (Kaasinen et al., 2019). Other studies have shown that different quantitative indices of DAT PET imaging—including the specific binding ratio, the putamen-to-caudate ratio, and the asymmetry index—are capable of discriminating with high accuracy between PDs and non-synucleinopathy diseases (Georgiopoulos et al., 2015; Iwabuchi et al., 2019). However, the clinical utility of the indexes in distinguishing between different PDs remains questionable (Eerola et al., 2005; Georgiopoulos et al., 2015; Buchert et al., 2019). In our study, subregion-to-subregion comparison showed rare difference among PDs groups, while asymmetric indexes of anterior and middle putamen could distinguish between PD patients and the others on group level.

Recently, the application of a SVM diagnostic framework to ^123^I-FP-CIT PET data by Nicastro et al. (2019) led to an accuracy for binary classification of PDs ranging between 62.9 and 83.7%. The accuracy values for binary and multiclass classification in this current study were as high as 82.2–89.7 and 80.4%, respectively, which were even in accordance with those reported in recent studies based on ^18^F-FDG PET imaging (Supplementary Table 1). Accordingly, additional features which had been detected by deep learning were integrated to achieve the relative high accuracy in the classification task.

In an effort to identify the most powerful training strategy, we tested a total of four different approaches in different combinations of patients with ES and AS disease—with the highest accuracy (77.8%) being observed for the combined ES+AS group. Notably, the accuracy in patients with AS was lower (55.6–58.3%) than that observed in patients with ES (69.4%). With the exception of sensitivity for the diagnosis of PSP, the ES+AS strategy performed better in terms of sensitivity, specificity, PPV, and NPV for each PDs subtype. These results suggest that DAT PET imaging of patients with AS markedly improves the classification results of patients with ES, while the opposite does not occur. We therefore believe that future replication efforts on the clinical utility of our computer-aided classification system should include both patients with ES and AS.

Another interesting finding from our study is that the greatest contribution to the classification process was attributable to the caudate and putamen, whereas the role of pallidum was negligible. Amongst the intranuclear subregions, the anterior caudate and the middle putamen contributed more prominently to the diagnostic classification. Notably, patients with idiopathic PD are characterized by an uneven pattern of intrastriatal dopaminergic loss (Kish et al., 1988) and differences in terms of intrastriatal dopaminergic loss may exist between different PDs (Ilgin et al., 1999; Knudsen et al., 2004). However, the addition of midbrain to our computer-aided classification system did not significantly improve accuracy (*p* = 0.683). These results may stem from a similar extent of midbrain dopaminergic neuron loss across different PDs, although published data in the field remain inconclusive (Hirsch et al., 1988; Fearnley and Lees, 1991; Martin-Bastida et al., 2017). An alternative explanation is that our computer-aided classification framework is unable to capture existing differences with respect to midbrain dopaminergic neuron loss, ultimately making their diagnostic contribution for distinguishing between different PDs not significant.

The present study has several limitations that merit comment. Firstly, the sample size is relatively small and validation studies on the data with larger sample size and from multiple centers are required. Secondly, the transparency of the whole pipeline could be compromised as a tradeoff of the accuracy when we extracted features for semantic segmentation via deep neural networks (Adadi and Berrada, 2018). The proposed pipeline should be further optimized to be more user-friendly and achieve better classification performance.

In conclusion, we described a user-friendly, semi-automatic computer-aided classification framework for PDs based on ^11^C-CFT PET imaging. The proposed approach is capable of extracting subtle image information and holds promise to improve the differential diagnosis of PD, MSA, and PSP in both research and clinical settings. However, replication in independent samples is paramount for ensuring external validity of our data.

## Data Availability Statement

The original contributions presented in the study are included in the article/Supplementary Material, further inquiries can be directed to the corresponding author/s.

## Ethics Statement

The studies involving human participants were reviewed and approved by Institutional Review Board at Huashan Hospital. The patients/participants provided their written informed consent to participate in this study.

## Author Contributions

XZ and PW: conception and design. JWu, JWa, PW, CZ, QX, and LiL: data acquisition. JX, SL, XZ, LeL, T-CY, QX, CZ, and PW: data analysis and interpretation. JX, SL, and QX: drafting the manuscript. PW, XZ, and CZ: review and critique. All authors contributed to the article and approved the submitted version.

## Funding

The work was supported by grants from the National Natural Science Foundation of China (61971142, 62111530195, 62011540404, 81771483, 81671239, 81971641, and 81361120393), the Research Project of Shanghai Health Commission (2020YJZX0111), the Clinical Research Plan of SHDC (SHDC2020CR1038B), the Shanghai Municipal Science and Technology Major Project (2017SHZDZX01), Youth Medical Talents-Medical Imaging Practitioner Program funded by Shanghai Municipal Health Commission and Shanghai Medical and Health Development Foundation [SHWRS (2020)_087], and the Fujian Province Joint Funds for the Innovation of Science and Technology (2019Y9070).

## Conflict of Interest

The authors declare that the research was conducted in the absence of any commercial or financial relationships that could be construed as a potential conflict of interest.

## Publisher's Note

All claims expressed in this article are solely those of the authors and do not necessarily represent those of their affiliated organizations, or those of the publisher, the editors and the reviewers. Any product that may be evaluated in this article, or claim that may be made by its manufacturer, is not guaranteed or endorsed by the publisher.
