# Supplementary material for: Computer-Aided Classification Framework of Parkinsonian Disorders Using 11C-CFT PET Imaging
Source: Front Aging Neurosci. 2022 Feb 1;13:792951. doi: 10.3389/fnagi.2021.792951 (PMC8846284; doi:10.3389/fnagi.2021.792951)
Supplement: Supplementary file 1 [file Table_1.DOCX]

**Supplementary materials**

There was a number of previous studies assessing the utility of ^18^F-FDG PET in the differential diagnosis of parkinsonian disorders. Different methods were proposed, e.g. the Statistic parametric mapping(SPM) supported reading methods (Eckert et al., 2005), the relevance vector machine approach (Garraux et al., 2013), the logistic regression algorithm (Tang et al., 2010; Tripathi et al., 2016), the hybrid method combines spatial covariance analysis and metabolic activity of disease-sensitive anatomic regions (Wu et al., 2016), as well as deep learning-based methods (Wu et al., 2018; Zhao et al., 2019). Comparation between some of the mentioned works of ^18^F-FDG PET and our work of ^11^C-CFT PET are shown in the table. Note that we only considered the accuracy with the three interested disorders, i.e., PD, MSA-P and PSP, and ignored other categories if any.

| Works | Dataset | Accuracy | Major Group | Sensitivity / Specificity | PPV / NPV |
| --- | --- | --- | --- | --- | --- |
| Eckert et al.  (SPM supported reading) 2015 | 76 PD vs. 24 MSA vs. 20 PSP | 96.67% | PD | 98.68% / 93.18% | 96.15% / 97.62% |
|  |  |  | MSA | 100.00% /100.00% | 100.00%/100.00% |
|  |  |  | PSP | 85.00% / 99.00% | 94.44% / 97.06% |
| Garraux et al.  2013 | ﻿42 PD vs. 78 APS | 84.23% | PD | 92.86% / 83.33% | 75.00% / 95.59% |
|  | ﻿42 PD vs. 27 MSA vs. 21 PSP | 75.00% | PD | 95.00% / 83.33% | 82.61% / 95.24% |
|  |  |  | MSA | 51.85% / 90.16% | 70.00% / 80.88% |
|  |  |  | PSP | 66.67% / 88.06% | 63.64% / 89.39% |
| Tripathi et al., 2016 | 96PD vs. 41MSA vs. 30PSP | 89.52% | PD | 95.71% / 91.43% | 95.71% / 91.43% |
|  |  |  | MSA | 64.71% / 98.86% | 91.67% / 93.55% |
|  |  |  | PSP | 88.89% / 91.95% | 69.57% / 97.56% |
| Y. Zhao et al., 2019 | 303PD vs. 155MSA vs. 84PSP | 95.20% | PD | 97.68% / 94.14% | 99.48% / 96.98% |
|  |  |  | MSA | 96.77% / 99.48% | 98.68% / 98.71% |
|  |  |  | PSP | 83.33% / 97.81% | 87.50% / 96.97% |
| Ours  (^11^C-CFT PET) | 50 PD vs. 57 APS | 85.00% | PD | 84.00% / 86.00% | 86.00% / 84.00% |
|  | 50 PD vs. 37 MSA vs. 20 PSP | 80.37% | PD | 86.00% / 84.21% | 82.69% / 87.27% |
|  |  |  | MSA | 78.38% / 88.57% | 78.38% / 88.57% |
|  |  |  | PSP | 70.00% / 95.40% | 77.78% / 93.26% |

# Reference

Eckert, T., Barnes, A., Dhawan, V., Frucht, S., Gordon, M.F., Feigin, A.S., Eidelberg, D., 2005. FDG PET in the differential diagnosis of parkinsonian disorders. Neuroimage 26, 912–921. https://doi.org/10.1016/j.neuroimage.2005.03.012

Garraux, G., Phillips, C., Schrouff, J., Kreisler, A., Lemaire, C., Degueldre, C., Delcour, C., Hustinx, R., Luxen, A., Destée, A., Salmon, E., 2013. Multiclass classification of FDG PET scans for the distinction between Parkinson’s disease and atypical parkinsonian syndromes. NeuroImage Clin. 2, 883–893. https://doi.org/10.1016/j.nicl.2013.06.004

Tang, C.C., Poston, K.L., Eckert, T., Feigin, A., Frucht, S., Gudesblatt, M., Dhawan, V., Lesser, M., Vonsattel, J.P., Fahn, S., Eidelberg, D., 2010. Differential diagnosis of parkinsonism: a metabolic imaging study using pattern analysis. Lancet Neurol. 9, 149–158. https://doi.org/10.1016/S1474-4422(10)70002-8

Tripathi, M., Tang, C.C., Feigin, A., De Lucia, I., Nazem, A., Dhawan, V., Eidelberg, D., 2016. Automated differential diagnosis of early parkinsonism using metabolic brain networks: a validation study. J. Nucl. Med. 57, 60–66.

Wu, P., Cheng, S., Wu, J., Stefan, F.Ã., Shi, K., Zuo, C., others, 2016. A new hybrid method for differential diagnosis of parkinsonism. J. Nucl. Med. 57, 1836.

Wu, P., Roy, A.G., Yakushev, I., Li, R., Conjeti, S., Ziegler, S., Wang, J., Forster, S., Navab, N., Schwaiger, M., others, 2018. Deep Learning on 18F-FDG PET Imaging for Differential Diagnosis of Parkinsonian Syndromes. J. Nucl. Med. 59, 624.

Zhao, Y., Wu, P., Wang, J., Li, H., Navab, N., Yakushev, I., Weber, W., Schwaiger, M., Huang, S.-C., Cumming, P., others, 2019. A 3D Deep Residual Convolutional Neural Network for Differential Diagnosis of Parkinsonian Syndromes on 18 F-FDG PET Images, in: 2019 41st Annual International Conference of the IEEE Engineering in Medicine and Biology Society (EMBC). pp. 3531–3534.
